# Failure dynamics of the global risk network

**DOI:** 10.1038/srep10998

**Published:** 2015-06-18

**Authors:** Boleslaw K. Szymanski, Xin Lin, Andrea Asztalos, Sameet Sreenivasan

**Affiliations:** 1 Social and Cognitive Networks Academic Research Center, Rensselaer Polytechnic Institute, Troy NY 12180; 2 Dept. of Computer Science, RPI, 110 8th Street, Troy, NY 12180; 3 Dept. of Computer Science & Management, Wroclaw University of Technology, 50-370 Wroclaw, Poland; 4 Dept. of Physics, Applied Physics and Astronomy, RPI, 110 8th Street, Troy, NY 12180.

## Abstract

Risks threatening modern societies form an intricately interconnected network that often underlies crisis situations. Yet, little is known about how risk materializations in distinct domains influence each other. Here we present an approach in which expert assessments of likelihoods and influence of risks underlie a quantitative model of the global risk network dynamics. The modeled risks range from environmental to economic and technological, and include difficult to quantify risks, such as geo-political and social. Using the maximum likelihood estimation, we find the optimal model parameters and demonstrate that the model including network effects significantly outperforms the others, uncovering full value of the expert collected data. We analyze the model dynamics and study its resilience and stability. Our findings include such risk properties as contagion potential, persistence, roles in cascades of failures and the identity of risks most detrimental to system stability. The model provides quantitative means for measuring the adverse effects of risk interdependencies and the materialization of risks in the network.

Modern society relies heavily on the robust functioning of systems that are intricately networked with one another, in an explicit and/or an implicit manner. While increasing the interconnectivity between infrastructure systems can result in a higher efficiency of service, it also makes the constituent systems vulnerable as a whole to cascading failures. Such cascades of failures have been studied generally in model networks^1^,^2^,^3^,^4^,^5^,^6^ and specifically in the context of engineered systems such as the power-grid^7^, the internet^8^ and transportation and infrastructure systems^9^, in the context of financial institutions^10^,^11^,^12^,^13^,^14^, and within ecological systems^15^. However, in addition to the risk of cascading failures being present within a particular domain (e.g., the network of financial institutions), there are also risks arising because of the coupling between systems in diverse domains^16^,^17^. Indeed, the primary thesis behind many societal collapses in the history of mankind is that of a cascade of diverse risks being materialized^18^. Examples of such cross-domain failure cascades include the collapse of the society on Easter Island stemming from deforestation that led to agricultural and economic instabilities and civil unrest, and the demise of the populations of the Pitcairn and Henderson Islands caused by an environmental catastrophe of their common but geographically distant trading partner of Mangareva. The acceleration of technological advances over the last two centuries and the virtual dissolution of geographical borders resulted in tightening of the coupling between risks in diverse domains and across geographically distant systems. The recent economic crisis and its widespread effects across the globe have demonstrated this all too clearly. Hence, there is an urgent need to quantify the dynamics of large scale risk materialization lurking within this globally interconnected tapestry. Moreover, enriching our understanding of systems formed by diverse, interconnected sub-systems spanning environmental, social, and infrastructural domains is an important component of the scientific study of the physical world.

As a qualitative means to this end, the World Economic Forum (WEF) publishes each year a report defining the network of global risks^19^. The report published in 2013 contains data on the likelihood of materialization of global risks, influence of risk materialization on other risks, and the potential impact that materialization of each risk has on the global economy. This data was prepared by over 1000 experts from government, industry, and academia.

The risks defined in the report are classified into five categories: economic, environmental, geopolitical, societal, and technological. This global risk network dataset thus provides an experts’ perspective on the threat of different risk materializations and their influence on other risks, both of which are often intangible to a non-expert.

The collection of evaluations made by large groups of experts is often termed crowdsourcing. Its value has been well documented through the rise of online encyclopedias and question-answer sites, and the use of tagging or classification by groups of experts for recommendation-based services such as Pandora and Netflix. Consequently here, we use the WEF global risk dataset as a starting point to performing a quantitative study that can generate actionable insights. Specifically, we propose a model for the materialization of risks on the network, in which internal (self-materialization) and external (contagion) risk materializations are separated using historical data. This allows us to estimate the parameters associated with the network, and thereby to define the dynamics of global risk network. Furthermore, we show that a model incorporating the influence of a risk’s network neighborhood on its own materialization matches past data on risk materialization better than a model which is oblivious to network effects. We analyze the model dynamics and study its resilience, stability, and risks contagion potential, persistence, and roles in cascades of failures, identifying risks most detrimental to system stability. The model provides quantitative means for measuring the adverse effects of risk interdependence and the materialization of risks in the network. Such quantitative insights can, in turn, form a sound basis for specific recommendations from domain experts on how to decouple the severely harmful risks from the network, or to reduce their materialization probabilities.

## Results

### Dataset

We utilize two datasets for this study, the first of which is a part of the WEF Report on Global Risks released in 2013 and available at^20^. The report defines *N* = 50 global risks (see^19^ for their detailed definitions) that are systemic, that is they constitute “breakdown in an entire system, as opposed to breakdowns in individual parts and components”^21^. As pointed out in^17^, each of these risks is a network itself. Together they represent a network of networks that are prone to catastrophic cascades of failures^3^. These failures are binary in nature, since they either manifest themselves or not (even though the size, range and economic impact of each manifestation may vary from one manifestation to another). The WEF Report on Global Risks^19^ includes expert assessments of risk materialization liklihoods and influence on other risks. Each expert defines these likelihoods by numerical value ranging from 1 (lowest likelihood) to 5 (highest likelihood) using integers and mid-points between integers within this range. Thus, although not explicitly stated, the assessment of risk materialization likelihoods by the experts was done on a quantitative scale with a resolution corresponding to probability increments of 1/8.

The averages and standard deviations of the expert likelihood values for each risk are also provided in the dataset. Thanks to high quality and diversity of the experts participating in this crowdsourced assessment, the margins of errors are small. This is demonstrated in Table 3 of Appendix 2, (p. 66) of^19^ that lists the average likelihood and impact scores and shows that their margins of errors (based on a 95% confidence level) vary from 1% to 2% of the average score values. The detailed analysis of the data collected in the dataset is provided in Appendices 1 and 2 of^19^.

We denote the average of assessments for risk *i* by *L*_*i*_. The dataset provides a list of pairs of risks, aggregated from expert selections of up to ten most influential pairs, such that both risks in each pair are perceived as having influence on each other. The weight of influence for each pair of the selected nodes is also listed and it represents the number of experts that chose the corresponding pair as influential. In the risk network, the selected pairs are represented as connected. We selected the model which uses just binary value representing whether two nodes are connected or not since this information yielded the same maximum expectation when optimized using historical data as the models that use the weights of connections. Accordingly, we designate a binary variable *b*_*ij*_ to each pair of risks (*i*, *j*) that is 1 if the pair appears at least once in the concatenated lists prepared by the experts, and 0 otherwise. Thus, *b*_*ij*_ captures some overall *association* between risks *i* and *j*. By definition *b*_*ij*_ = *b*_*ji*_. There are 515 bidirectional edges defined that way, thus, the average degree of each node is relatively high, 20.6 edges per node.

**Figure 1:**
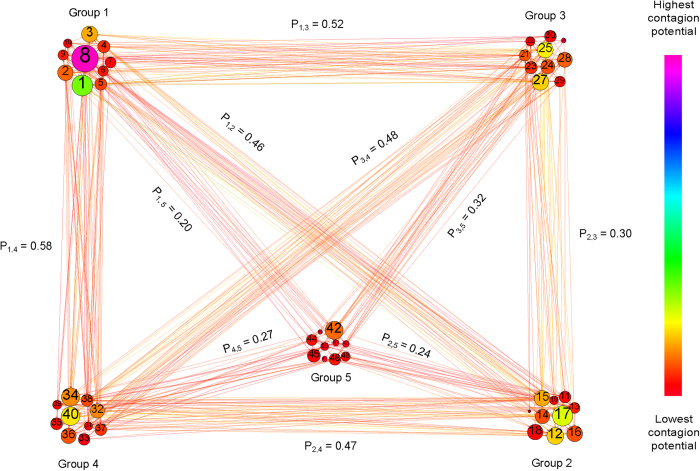
Connectivity of Risk Groups: Visualization of inter-group connectivity where node’s color corresponds to its total connectivity and the numbers of lines connecting the groups indicate strength of the inter-group connectivity. Groups 1 (economic risks), 2 (environmental risks), and 3 (geopolitical risks) are the best connected, so risks from these groups dominate the list of most persistent nodes. The remaining two groups: 4 (societal risks) and 5 (technological risks), have fewer connections to other groups. The inter-group edges are labeled with probability of inter-group connections (intra-group edges are shown in Fig. 2).

**Figure 2:**
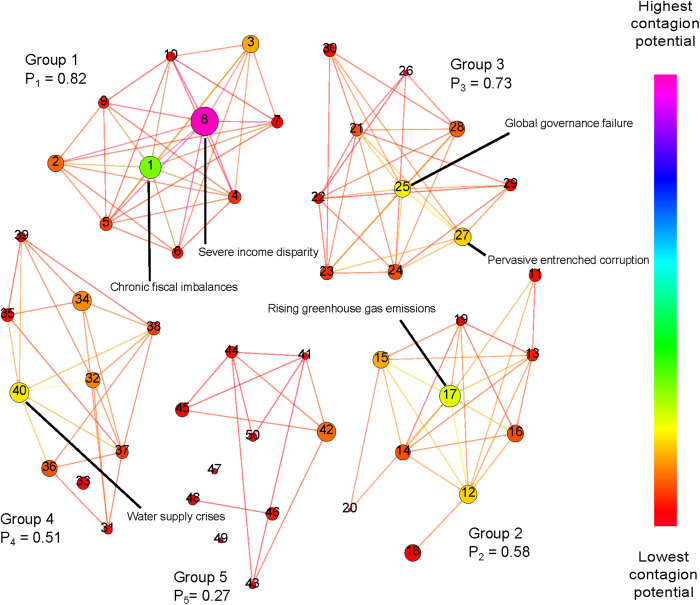
Global risk network intra-group connectivity and nodes congestion potentials derived from optimal model parameters: Each node is sized proportionally to its internal failure probability while node color corresponds to its total contagion potential. The number of edges in each group shows the intra-group connectivity. The nodes with the highest congestion potential are identified by name and include risks 8 “Severe income disparity”, 17 “Rising greenhouse gas emissions”, 1 “Chronic fiscal imbalances”, 40 “Water supply crises”, 25 “Global governance failure” and 27 “Pervasive entrenched corruption”.

The entire global risk graph can be modeled as a Stochastic Block Model^22^ with probability *p*_*g*_ of the edge existing between two risks in the same group *g* (this probability differs from group to group) while connections between nodes from two different risk groups, (*g*1, *g*2), are drawn with unique probability *p*_*g*1,*g*2_. The values of these probabilities for the WEF global risk graph are shown in Figs. 1 and 2. The adjacency matrix of this graph is denoted *A*, and its binary element *a*_*i*,*j*_ is 1 if and only if edge (*i*, *j*) exists. We express the probabilities of risk materialization in terms of the *L*_*i*_s and *b*_*ij*_s obtained from the WEF dataset and the parameters of our model.

In order to optimize parameters for our model, we require a time series of risk materialization events over a contiguous period. We collected data on the materialization of each of the 50 risks over the period 2000–2012 by systematically surveying news, magazine and academic articles, and websites identified using targeted queries on Google, as well as Wikipedia entries pertinent to specific risk materialization events. For brevity, we refer to this dataset as the *historical* dataset. Overall, it provides 50 × 156 = 7,800 data points for tuning the system parameters.

### Model

The failure dynamics of the global risk network can be modeled using Alternating Renewal Processes (ARP)^23^, which were initially used for engineered systems, but recently have been applied to science and economic applications^24^. Nodes under ARP alternate between normal state and failure state, and the corresponding events of failure activation and recovery are responsible for the state transitions. Typically, these events are assumed to be triggered by the non-homogeneous Poisson Processes (i.e. Poisson Processes with time dependent intensities)^23^. In our systems, use of the Poisson distribution is justified because each failure represents a systemic risk that can be triggered by many elementary events distributed all over the globe. Such triggering distorts any local temporary patterns of events (such as periodic weather related local disasters in some regions of the globe). Moreover, the time-dependence of intensity of risk activation is the results of influence that active risks exert on passive risks connected to them. Accordingly, each risk at time *t* is either in state 1 (materialized or *active*) or state 0 (not materialized or *inactive*).

In traditional ARP, there are two directly observable processes, one of risk activation and the other of recovery from the active risk. However, in our model, we introduce two latent processes that together represent the risk activation process. As explained later, we use the Expectation-Maximization Algorithm^25^ to find model parameters that make the model optimally match historical data. Consequently, we assume that changes in the state of each risk result from events generated by three types of Poisson processes. First, for a risk *i*, given that it is in state 0, its *spontaneous* or *internal* materialization is a Poisson process with intensity 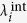. Similarly, given the risk in state 1, its recovery from this state, and therefore transition to state 0, is a Poisson process with intensity 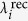. Finally, given that risks *i* is in state 0, and *j* in state 1 are connected, the materialization of risk *i* due to the *external* influence of risk *j* is a Poisson process with intensity 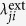. We assume that each of these processes is independent of each other. We also evaluated models in which recovery is represented by two latent processes, one of internal recovery and the other of recovery induced by either the connected passive or active risks. In both cases, the optimal intensity of the externally induced recovery was 0. Thus, the simpler model with just internal recovery is used as it yields the same results as the more complex models using latent processes for recovery.

The model is similar to a model of house fires in a city, where some houses burn alone from a self-started fire, but others are ignited by the burning neighboring houses. Yet, the recovery, in this case rebuilding of a burnt house, is independent of the state of its neighboring houses.

Denoting by *s*_*i*_(*t*) the probability at time *t* that the state of a risk *i* at that time is 1, we can express the expected number of risks materialized at time *t* as the sum of all *s*_*i*_(*t*)‘s, each of which is defined by the following Ordinary Differential Equation (ODE):


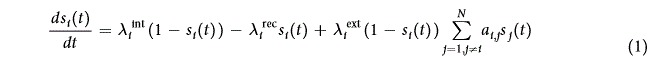


Checking stability, we conclude that this system of non-linear ODEs has only one unique stable point in the feasible range 0 ≤ *s*_*i*_(*t*) ≤ 1, which can easily be found numerically. Moreover, this system of ODEs for a fully connected graph when the intensities *λ*_*s*_, *λ*_*r*_, *λ*_*e*_ of the three Poisson processes are independent of the node on which they operate, and for all nodes starting in the same initial condition *s*(0) has the analytic solution of the form 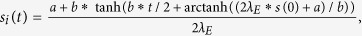 where *λ*_*E*_ = (*n* − 1)*λ*_*e*_, *a* = *λ*_*s*_ + *λ*_*r*_ − *λ*_*E*_, and 
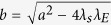
. This solution tends asymptotically to 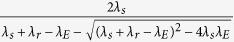.

For nearly all events that we consider here, it is difficult to assign precise starting and ending times for their periods of materialization. Thus, it is more proper to consider a Bernoulli process in which the time unit (and also time step of the model evolution) is one calendar month (we ignore the minor numerical imprecision arising from the fact that calendar months have different numbers of days). Consequently, all events starting in the same month are considered to be starting simultaneously. Hence, at each time step *t*, each risk *i* is associated with a binary state variable *S*_*i*_(*t*) ∈ {0,1}. The state of the entire set of risks at time *t* can therefore be represented by a state vector 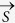
(*t*). Thus, the dynamics progresses by assuming that at each time step *t* > 0:

1. a risk *i* that was inactive at time *t* − 1 materializes internally with probability 
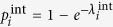
.
2. a risk *j* that was active at time *t* − 1 causes a neighboring risk *i* that was inactive at time *t* − 1 to materialize with probability 
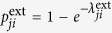
.
3. a risk *i* that was active at time *t* − 1 continues its materialization with probability 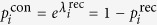.

It is easy to show that for real time *t* measured in finite time units (months in our case), the Poisson process assumption for events results in a probability 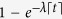 of an event happening in at most 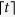 time units which is identical to the assumed Bernoulli process. The advantage of the latter process is that in each step the probability of event is known, simplifying maximum likelihood evaluation of the model parameters. Finally, the dynamics described above imply that the state of the system at time *t* depends only on its state at time *t* − 1, and therefore the evolution of the state vector 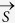
(*t*) is Markovian.

Given the probabilities of internal materialization, external influence, and continuation, that is just 1 minus the probability of recovery, the probability of a transition in a risk’s state between consecutive time steps can be written in terms of these probabilities:


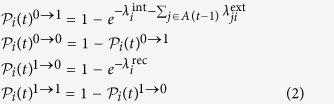


where *A*(*t*) represents the risks that are active at time *t*, and 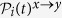 is the probability that risk *i* transitions between time *t* − 1 and *t* from state *x* to state *y*, or in other words *S*_*i*_(*t* − 1) = *x* and *S*_*i*_(*t*) = *y*.

The mapping of the Poisson process intensities into the expert assessments is described in the Methods section. Each of the probabilities of Bernoulli processes is mapped onto the probability obtained from expert assessment of likelihood of risk failure by single-parameter formula. We find the values of the model parameters that optimize the model match with the historical data, and we use expert assessments to individualize probabilities of Bernoulli processes for each risk. In essence, the expert assessments are defining those probabilities for each risk in relations to probabilities for other risks, while model parameters map performance of all risks onto historical data. By distinguishing between internal and external materialization factors, the mapping of parameters onto historical data enables us also to decompose risk materializations into these two categories. Once the mapping is done, the model is complete and can be used to evaluate global risk dynamics.

From several alternative models presented in Methods section, we discuss below the best performing network model which uses all three parameters, and the disconnected model which sets the value of probability of influence of a risk materialization on any other risk to zero, effectively isolating risks from each other.

### Contagion potentials of risks in the network model

Here, we investigate the relative importance of different risks. First, in analogy with epidemic studies, we calculate the *contagion potential* of individual risks, i.e., the mean number of materializations that a risk induces given that it alone has materialized. For risk *i*, the exact expression for this quantity is:


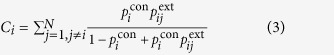


where *N* refers to the total number of risks. This expression assumes that risks other than *i* can only be activated through the influence of risk *i* and not internally.

Fig. 2 shows a visualization of the network capturing the contagion potentials as well as the internal failure probabilities in the network model (the mapping of node indices to the risks is provided in Table 1). As illustrated, the internal failure probability does not strictly show a positive correlation with contagion potential. Hence, a frequently materializing risk does not necessarily inflict the most harm to the system as a result of its influence on other risks. For example, although risk 42 - “Cyber attacks” has a relatively high probability of internal materialization, its contagion potential is low. In contrast, risk 25 - “Global governance failure” has both a high probability of internal materialization and a high contagion potential. However, most striking is the fact that risk 8 - “Severe income disparity” has both the highest internal materialization probability and the highest contagion potential. This is particularly notable in the light of recent claims that income disparity in the United States is the highest in over eight decades^26^.

The five risks with the highest contagion potentials are: 8 - “Severe income disparity”, 1 - “Chronic fiscal imbalances”, 17 - “Rising greenhouse gas emissions”, 40 - “Water supply crises”, and 12 - “Failure of climate change adaptation”. When ranked purely by raw likelihood values *L*_*i*_ (or equivalently by the internal failure probabilities 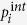), the only change is on the fifth position, where risk 12 is replaced by risk 34 - “Mismanagement of population ageing” which moves up from eleventh position to fifth.

### Network activity level and risk-persistence

**Figure 3:**
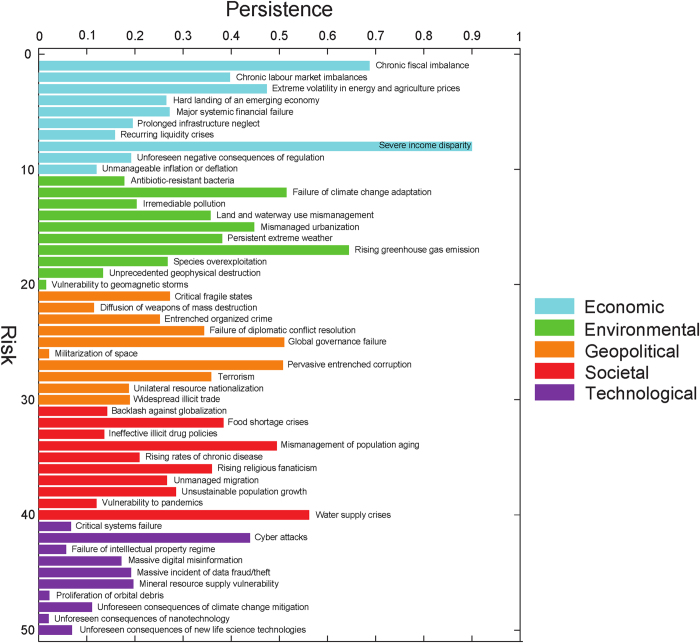
Persistence of risks in model simulation: The bar graph shows the overall fraction over 10^6^ simulated experiments, each consisting of 2200 time steps of which the initial 200 are ignored, that a given risk was active. The top five most persistent risks are 8 - “Severe income disparity”, 1 - “Chronic fiscal imbalances”, 17 - “Rising greenhouse gas emissions”, 40 - “Water supply crises”, and 12 - “Failure of climate change adaptation”. Not surprisingly this is the same lists as the list of the most contagious risks identified in the text because symmetric influence relationship makes them also most vulnerable to contagion.

Next, we perform Monte-Carlo simulations of both the network model and the disconnected model. Fig. 3 shows the fraction of time steps over 10^6^ simulations, each consisting 2200 time steps, during which a given risk was active (the initial transient consisting of 200 steps was ignored). We call this fraction the *persistence of the risk*. Each simulation was initiated with the same active risks that are present in the first month of historical data (i.e. January 2000). The most persistent risk was 8 active 90% of the time, followed by risk 1, active 68% of the time, risk 17, active 64% of the time, risk 40, active 56% of the time, and risk 12, active 51% of the time.

Another interesting aspect is the distribution of the number of active risks obtained in the simulation. The 10^th^ percentile value of the number of active risks is below 8, while the 90^th^ percentile value of the number of active risks is over 19, implying that about 80% of the time, the number of active risks will lie between these two values.

The steady state (long-time limit) activity levels indicate that the *carrying capacity* of the global risk network at the present time is 27% of the size of the network, i.e., about 13.8 risks are active all the time. The top seven risks observed to be active most frequently in simulations are 8, 1, 17, 40, 12, 25, and 27. These seven risks contribute on average 4.3 members to the total activity level at any month. This implies that other 43 risks together contribute on average the remaining 9.4 active risks, thus their activity level per risk is nearly three times lower than it is for the top seven risks.

### Cascades due to single risk materializations

**Figure 4:**
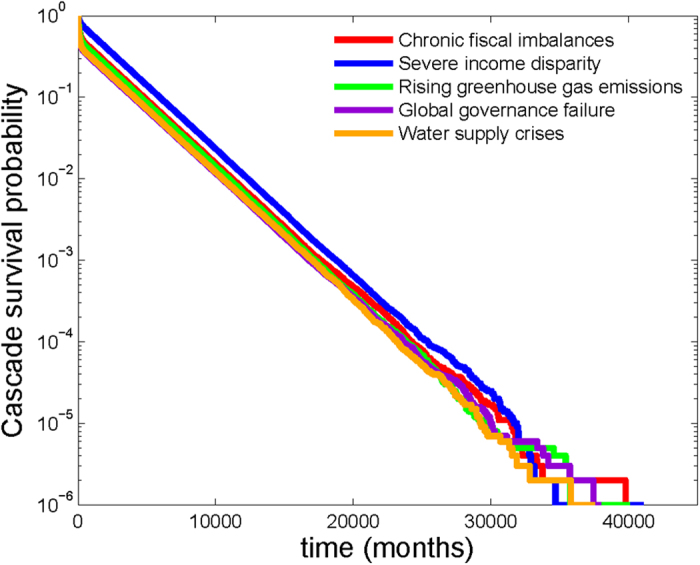
Survival probability of a risk cascade initiated by a single failure: Each curve shows the survival probability of a cascade initiated by a specific risk as a function of time. Five risks with high contagion potentials (listed in the text) were chosen as the respective cascade initiators, and the number of surviving cascade realizations among a total of 10^6^ realizations was computed for each chosen initiator. The straight line on the linear-logarithmic scale shows clear evidence of an exponentially decaying survival probability.

We further study the effect of risk interconnectivity by investigating the survival probability of a failure cascade initiated by a particular risk’s materialization. Specifically, we perform 10^6^ Monte-Carlo simulations of the model, each running for 50,000 time steps, starting with a given single risk active and setting the internal failure probabilities of all risks to zero. Thus, all subsequent risk materializations (after the initial one) are caused purely by the *cascade* of failures propagating within the network. Note that this is different from the true activity dynamics within the network discussed previously. These simulations are carried out to demonstrate the extent to which the connectivity between risks facilitates secondary activations. Fig. 4 shows the survival probabilities for cascades initiated by five highly contagious risks ranked in descending order of contagion potential. The linear nature of the curves on the linear-logarithmic scale indicates that survival probabilities decay exponentially with time. Despite that, even the cascade initiated by the *least* contagious risk in the displayed data, risk 40 “Water supply crisis”, has a greater than 1% chance of continuing beyond 10,000 months, i.e., over eight centuries. These long cascade lifetimes, even in the absence of internal failures, demonstrates the profound disadvantage of interconnectivity of global risks.

**Figure 5:**
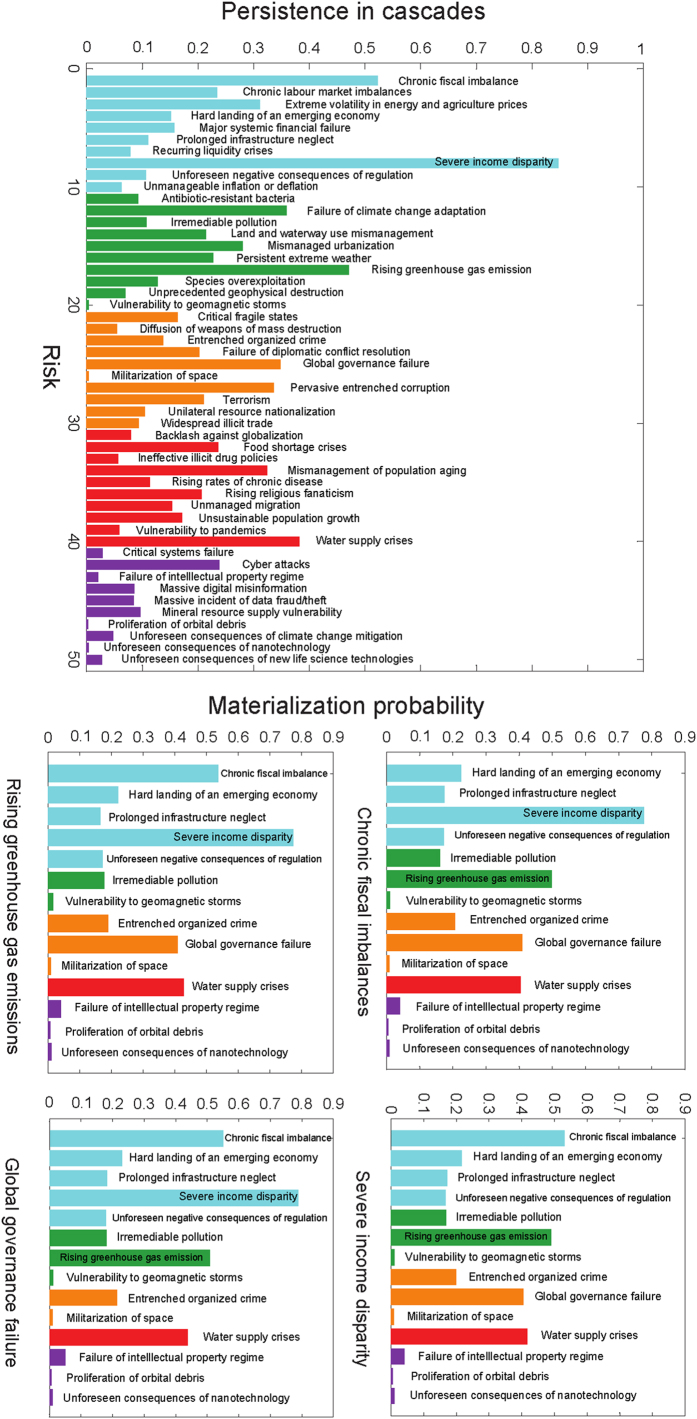
Persistence and materialization probabilities of risks in cascades: (**a**) The bar graph shows the fraction of the total lifetime of a cascade that a given risk is expected to be active, as obtained from 10^6^ simulations for each of 15 different initiators, where initiators are chosen from sets of risks with high contagion potential, medium contagion potential and low contagion potential. The specific risks chosen as initiators were risks 1,8,9,12,16,20,23,25,26,27,31,33,42,47,49. (**b**) The bar graphs show the materialization probabilities of four labeled risks, as a function of the initiator of the cascade (whose names are shown within their respective bars). Each experiment ended when either the selected risk was infected, or all risks became inactive.

Next, we investigate which risks are predominantly responsible for the cascades persisting for such long time-scales. Fig. 5 shows the expected fraction of the lifetime of a cascade for which a particular risk is active, in ranked order. The top five highest active risks are 8, active for 83% of the cascade lifetime, 1, active for 53% of the lifetime, 17, active for 46% of the lifetime, 40, active for 39% of the lifetime, and 12, active for 35% of the lifetime. Interestingly, the lists of top five most persistent risks observed in the cascades and seen in the full dynamics of activation (when all nodes undergo both internal and external activation Poisson processes) are identical.

We also compute the probability that the cascade resulting from the materialization of a given initiator risk would result in the materialization of a selected risk. Specifically, we consider the probability of materialization of the four risks, 8, 1, 17 and 25, observed to be among the top five risks most frequently active in simulations. Risk 8 is the initiator that yields the highest materialization probabilities for risks 1, 17, and 40, while it materializes with highest probability when the initiator risk is 1 and is followed by risks 17, 25, and 40. The risks 8 materializes also with highest probabilities for initiators 17 and 25 but then it is followed by risk 1.

### Model dynamics

In this section, we compare the dynamics of the network and disconnected models. In the latter model, risks change their states only through internal Poisson processes of materialization and recovery. Thus, the dynamics of this model is simple, the activity level oscillates around the sum of average activity levels of all risks. For risk *i* this quantity, *a*_*i*_, is defined as


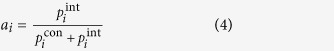


The resulting average activity level for the disconnected model is 10.75. For comparison, the continuous time model is defined by the set of the following linear ODEs:


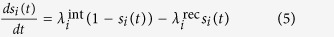


that has a simple solution: 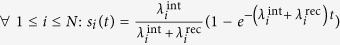. The sum of these solution functions represents the average activity level of the model. It asymptotically tends to 
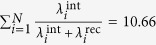
, in good agreement with the discrete time Bernoulli process model.

In the network model activity level depends on the current set of active risks, as active risks influence inactive ones. If currently active risks have low contagion potential, the system will drift to the lower activity level. Analyzing this case shows that this drift can cause all risks to be inactive. On the other hand, active risks with high contagion potential will cause the system to even higher activity level than currently attained. Up to top 20 risks with the highest contagion potential will cause the drift towards increasing activity level. Thus, the network model will oscillate between the upper bound of 21 active risks and a natural lower bound of having all risks inactive (albeit the latter is less likely than former, because highly contagious risks are also highly vulnerable to materialization via contagion). The average activity level for the network model is 13.79, which is close to the value of 13.94 yielded by the continuous time model defined by Eq. (1). The distribution of activity levels in both models with discrete time is close to normal, *N*(13.79, 3.71) for network model and *N*(10.75, 2.69) for disconnected model.

## Methods

The first step towards defining the model is to relate the Poisson process intensities that determine the event probabilities in the model, to quantities provided by the expert assessments, namely, the likelihoods (*L*_*i*_) of internal materializations of risks over a decade, and the influence (*b*_*ij*_) that a given risk’s materialization has on other risks.

### Mapping experts’ assessments into Poisson process intensities

We first normalize the likelihood values to probabilities in their natural range of [0, 1] by a simple linear transformation:


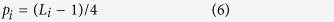


This normalized likelihood value *p*_*i*_ is in direct proportion to the expert assessment *L*_*i*_, and for our purposes captures the risk’s vulnerability to failure. Next, we assume that the relationship between the probability, 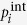, that a risk *i* materializes internally in a time unit (one calendar month) and this risk normalized likelihood value obeys the following polynomial form, with a parameter *α* defining the exact mapping:


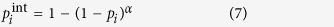


Thus, the probability of failing within a time period increases as the vulnerability *p*_*i*_ increases, and Eq. (7) coupled with our earlier assumption that the internal risk materialization is a Poisson process with intensity 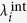, yields:


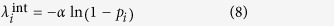


An advantage of Eqs. (7) and (8) is that their forms remain invariant under changes of the time-scale under consideration. Indeed, multiplying the original value of time unit by factor *f* simply changes the Poisson process intensity and the value of *α* by the same factor *f*. For example, for the time unit of the expert materialization likelihood assessment set to a decade, the corresponding value of *α* is 120 times larger than the value obtained when the time unit is set to a month. Moreover, as is seen in the following equations, the ratio of intensities is defined entirely by the ratio of the corresponding model parameters and is independent of the risk for which the corresponding Poisson processes generate events. So model parameters define the same ratio of intensities of all risks, while likelihood assessments define individual values of these intensities for each risk.

Another advantage of the form of Eq. (7) is that it can represent convex (for *α* > 1), linear (with *α* = 1), or concave (for *α* < 1) function, with the parameter *α* defining the shape that best matches a given set of historical data.

We adopt a similar reasoning to the mapping between the probability of continuation in a time unit 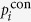 and the normalized likelihood values *p*_*i*_. We start with the assumption that the probability of a materialized risk continuing over a time unit is 1 − (1 − *p*_*i*_)^*γ*^, where parameter *γ* defines the mapping from likelihood to probability. This dependence captures the increasing likelihood of continuation as the vulnerability *p*_*i*_ increases and leads to the following equation:


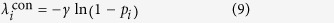


Finally, following similar arguments as above, the intensity 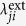 of the Poisson process that enables a materialized risk *j* to influence the materialization of risk *i* is a function of parameter *β* defined as:


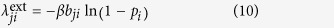


The factor *b*_*ji*_ on the right hand side merely serves to capture the fact that the risks *i*, *j* must be perceived by the experts as having an influence on each other, in order for the probability of influence to be non-zero.

The formulas provided in Eqs. (8), (9) and (10) define the model completely, and all that remains is to fit the parameters *α*, *β*, and *γ* optimally to the historical data capturing the risk materialization events over the last 13 years. In the historical dataset, each risk is assigned a state per month (the fundamental time unit) over the period of 2000–2012. Thus, the likelihood of observing this particular sequence of risk materialization events through the dynamics generated by our model can be written as:


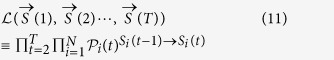


where *T* = 156 is the number of time units in the historical dataset and *N* = 50 is the number of risks. Consequently, the logarithm of the likelihood of observing the sequence is:


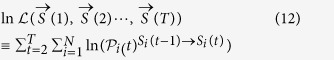


**Figure 6:**
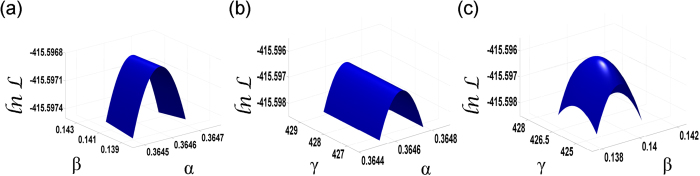
Log-likelihood of data as a function of model parameters: Behavior of the log-likelihood of observing the historical sequence of risk materialization events as a function of pairs of model parameters. The three plots show the variation of 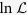 for fixed *β*, *γ* and *α* respectively as a function of the values of the two remaining parameters. The first plot has *γ* fixed at its optimal value of 427, the second plot has *β* fixed at 0.14 and the last plot has *α* fixed at 0.365.

Following the well-known process of maximum likelihood estimation^25^,^27^, we find the arguments that maximize the log-likelihood to optimize the model fitness. For a given set of values of parameters *α*, *β*, and *γ*, one can compute the log-likelihood of observing the given time-series of risk materialization using Eqs. (2) and (12). Thus, by scanning different combinations of *α*, *β*, and *γ* over their respective feasible ranges, and by computing the resulting log-likelihoods, one can find with the desired precision the values of *α*, *β*, and *γ* that maximize the likelihood of observing the data. The likelihood function is smooth (see the plot in Fig. 6) with a unique maximum that guarantees that found parameter values are indeed globally optimal for the model considered. With the time unit of a decade, these optimal values (marked by ^*^ superscript) are α^*^ = 0.365 ≈ 4/11,β^*^ = 0.14 ≈ 1/7,γ^*^ = 427, and the log-likelihood of observing the data given these parameters is −415.6. We refer to so-defined model as *network model*.

### Establishing model properties

Next, we measure how vulnerable our model is to noise in the expert data. To do this, we randomly perturb each average likelihood value provided by the experts to a value within one standard deviation from the average, and create 10 sets of such randomly perturbed likelihood data. Next, we compute 10 parameter sets that maximize log-likelihood of observing the historical data. Then, we run 10 models, termed *noisy data models*, defined by the obtained parameter sets. For each noisy data model, we compute its monthly activity level, which is the number of risks active in each month averaged over 10^6^ runs of this model. Finally, we compute the maximum differences between parameters and monthly activity levels at each month of the network model and all 10 noisy data models. The optimal parameters of noisy data models were within ±1.4% of those values for network model. Average activity levels of these models were within ±3% of the corresponding value for the network model. Finally, the maximum log-likelihoods of noisy data models are within ±1% of this value for network model. These are small differences compared to the corresponding differences between the network and others models discussed below.

### Alternative models

We measure the importance of network effects by comparing the maximum log-likelihood obtained above to the corresponding maximum log-likelihood value for a model which is oblivious to network effects, so in which *β* = 0. We refer to this model as the *disconnected model*. With the time unit of a decade (which experts used in their likelihood assessment), the two optimal parameters are *α*^*d*^ = 0.91, *γ*^*d*^ = γ^*^ = 427 and maximum log-likelihood is -420.1. Using the likelihood ratio (LR) test^28^, we conclude the network model outperforms the disconnected model at a significance level of 0.01. This result demonstrates that to fully uncover the value of expert data requires accounting for network effects, as we have done in our network model.

Setting *α* = 1.0 creates a model that we refer to as *expert data based model* which yields maximum log-likelihood that is only slightly lower (by 0.02%) than for the optimal disconnected model. More importantly, it results in a particularly simple form for one-decade risk *i* materialization probability: 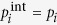. This linear mapping demonstrates that the averages of experts’ assessments of risk materialization likelihoods are in fact excellent estimates of probabilities of risk failures in the ten-year period. It also attests to validity of our historical data and of expert assessments, since any mistake in those two datasets would make a mapping from expert data to probabilities a complex function. Similar high quality expert forecast in strategic intelligence was discussed in^29^. Yet, this result uncovers the limit of expert assessments, as they capture the aggregate probability of failures resulting from internal and external risk materializations without ability to distinguish between them. Since external materialization depends on which risks are active, any state transitions of risks change such aggregate probability. Our model, through parameter mapping onto the historical data, is able to separate external and internal materialization probabilities and therefore is valid regardless of the current or future states of the risks.

We also evaluate the value of experts’ assessments of risks susceptibility to failures and their influence on each other for modeling risks. An alternative model with individual parameters for each risk susceptibility and influence would have too many degrees of freedom to be well-defined. However, the network model applied to risks with uniform likelihood and influence, a model to which we refer to as *uniform model*, and which is therefore agnostic to expert data, yields a maximum log-likelihood of -437.1, far from what the disconnected and expert data based models deliver. According to the LR test^28^, the disconnected and expert data based models cannot be distinguished from each other with any reasonable significance level. However, the same test allows us to conclude that these two models outperform a simple uniform model based only on historical data with a significance level of 0.001.

Summary of models discussed here is provided in Table 2.

## Discussion

To summarize, in this study we have presented a method of obtaining a quantitative picture of the global risk network, starting from the qualitative observations provided by 1000 WEF experts. We assume a three parameter network model for the propagation of risk materialization (representing the corresponding network node failures), and obtain maximum likelihood values for the parameters using historical data on risk materialization.

Our model was built upon expert assessments available in the WEF report which enabled the construction of a detailed and heterogeneous weighted network of risks. As we show, ignoring network effects (i.e. the disconnected model) or ignoring specific heterogeneities in the failure likelihoods and influence (i.e. the uniform model) yielded poor results in comparison to the network model. This underscores the importance of the expert assessments in building a model capable of matching the available activity data well, and therefore yielding reliable insights. We have also found the greatest strength of expert assessments, which is nearly perfect forecast of aggregate failure probabilities of different risks, but also those assessments greatest weakness, which is inability to separate external risk materialization probabilities form internal ones. We have developed an approach in which by selecting proper model parameters and using maximum likelihood estimation to find optimal model parameters, we are able to do such separation.

We have uncovered the global risk network dynamics and measured its resilience, stability, and risks contagion potential, persistence, and roles in cascades of failures. We have identified risks most detrimental to system stability and measured the adverse effects of risk interdependence to the materialization of risks in the network. According to these studies, the most detrimental is risk 8 -“Severe income disparity”. Other risks that play a dominant role due to either their contagion potential or their persistent materialization are: 1 - “Chronic fiscal imbalances”, 25 - “Global governance failure”, 27 - “Pervasive entrenched corruption”, 12 - “Failure of climate change adaptation”, 17 - “Rising greenhouse gas emissions”, and 40 - “Water supply crises”.

Utilizing the complete network model generated using the WEF data provides a much more detailed picture of the threats posed by different risks than the one obtained by simply relaying only on their failure-likelihood *L*_*i*_ values and using the disconnected model. Additionally, our analysis demonstrates that the carrying capacity of the network, i.e. the typical activity expected in the network given the current parameters, is about 13.7 risks or 27% of the total number of network nodes, of which four are persistently chosen from a subset of seven risks (see Fig. 3). Aiming to reduce this overall carrying capacity could potentially be an overarching goal of global risk minimization.

There are several prospects for extending the model that we presented here and its further analysis. First, obtaining more robust historical estimates of risk materialization may help us improve the fitting of the model. Secondly, it will be beneficial to account for slow evolution of network parameters in time. This change in network characterization will be captured by a model through expert data provided in yearly WEF reports, resulting in time dependent *L*_*i*_s and *b*_*ij*_s. Furthermore, the accuracy of the model could possibly be improved by assuming the existence of different dynamics for chronic risks as compared to sporadic risks.

From a larger perspective, our attempt here has been to utilize data crowdsourced from experts towards gaining a quantitative picture of the network of global risks, which in turn has yielded some actionable insights. The network by definition has risks of varying complexity, which arguably makes the risk mitigation process more involved for some risks than for others. In such a scenario, our quantification of the relative impacts of different risks could provide a valuable guide to any cost-benefit analysis involved in the design of policies or strategies aimed at global risk minimization.

The ideal next step given the insights provided by our model would be for domain experts to provide tailor-made recommendations for the pertinent risks, such that the likelihood of systemic failures is strongly curbed. The effect of such recommendations can also be tested using our model. We therefore believe that the contribution of this paper is to implement the crucial step that lies between the gathering of crowdsourced data and the prescription of domain-specific recommendations.

## Additional Information

**How to cite this article**: Szymanski, B. K. *et al.* Failure dynamics of the global risk network. *Sci. Rep.* **5**, 10998; doi: 10.1038/srep10998 (2015).

## Figures and Tables

**Table 1:** Mapping of indices to risks used throughout the paper.

ID	Risk	ID	Risk
1	Chronic fiscal imbalances	2	Chronic labour market imbalances
3	Extreme volatility in energy and agriculture prices	4	Hard landing of an emerging economy
5	Major systemic financial failure	6	Prolonged infrastructure neglect
7	Recurring liquidity crises	8	Severe income disparity
9	Unforeseen negative consequences of regulation	10	Unmanageable inflation or deflation
11	Antibiotic-resistant bacteria	12	Failure of climate change adaptation
13	Irremediable pollution	14	Land and waterway use mismanagement
15	Mismanaged urbanization	16	Persistent extreme weather
17	Rising greenhouse gas emissions	18	Species overexploitation
19	Unprecedented geophysical destruction	20	Vulnerability to geomagnetic storms
21	Critical fragile states	22	Diffusion of weapons of mass destruction
23	Entrenched organized crime	24	Failure of diplomatic conflict resolution
25	Global governance failure	26	Militarization of space
27	Pervasive entrenched corruption	28	Terrorism
29	Unilateral resource nationalization	30	Widespread illicit trade
31	Backlash against globalization	32	Food shortage crises
33	Ineffective illicit drug policies	34	Mismanagement of population aging
35	Rising rates of chronic disease	36	Rising religious fanaticism
37	Unmanaged migration	38	Unsustainable population growth
39	Vulnerability to pandemics	40	Water supply crises
41	Critical systems failure	42	Cyber attacks
43	Failure of intellectual property regime	44	Massive digital misinformation
45	Massive incident of data fraud/theft	46	Mineral resource supply vulnerability
47	Proliferation of orbital debris	48	Unforeseen consequences of climate change mitigation
49	Unforeseen consequences of nanotechnology	50	Unforeseen consequences of new life science technologies

**Table 2:** Summary of models: Parameters for the models mentioned in the text, and the data utilized to estimate the respective parameters.

Model	Parameters	Data used
network model	*α*, *β*, *γ*	*L*_*i*_, *b*_*ji*_, historical data
disconnected model	*α*, *γ* (*β* = 0)	*L*_*i*_, historical data
expert data based model	*γ* (*α* = 1,*β* = 0)	*L*_*i*_, historical data
uniform model	*λ*^int^, *λ*^con^, *λ*^ext^	historical data

Parameters *α*, *β*, and *γ* govern the Poisson process intensities for internal materializations, pairwise influence, and continuation, as expressed in Eqs. (8), (10), and (9) respectively. *L*_*i*_ represents the likelihood score provided for risk *i* by the WEF report, and *b*_*ji*_ is a binary variable that adopts a value of 1 if the materialization of risk *j* is deemed to have an influence on the materialization of risk *i* in the opinion of at least one experts’. The expert data based model is the disconnected model in which value of parameter *α* is restricted to 1.0. The uniform model uses two parameters for the Poisson process intensities for internal materialization and materialization continuation (Eqs. (8) and (9)) which are assumed to be identical for all risks. It uses the third parameter to define the influence probability between risks (Eq. (10)) which is assumed to be the same for all risk pairs. The network model outperforms all other models in explaining the observed data with the significance level of 0.01 or lower.

## References

[b1] Watts D. J. A simple model of global cascades on random networks. P. Natl. Acad. Sci. USA 99, 5766–5771 (2002). 10.1073/pnas.082090499 PMC122850 16578874

[b2] Motter A. E. & Lai Y. C. Cascade-based attacks on complex networks. Phys. Rev. E 66, 065102 (2002). 10.1103/PhysRevE.66.065102 12513335

[b3] Buldyrev S. V., Parshani R., Paul G., Stanley H. E. & Havlin. S. Catastrophic cascade of failures in interdependent networks. Nature 464, 1025–1028 (2010). 20393559 10.1038/nature08932

[b4] Asztalos A., Sreenivasan. S., Szymanski. B. K. & Korniss G. Distributed flow optimization and cascading effects in weighted complex networks. Eur. Phys. J. B 85, 1–10 (2012).

[b5] Brummitt C. D., D’Souza R. M. & Leicht E. A. Suppressing cascades of load in interdependent networks. P. Natl. Acad. Sci. USA 109, E680–E689 (2012). 10.1073/pnas.1110586109 PMC3311366 22355144

[b6] Roukny T., Bersini H., Pirotte H., Caldarelli G. & Battiston S. Default cascades in complex networks: topology and systemic risk. Sci. Rep. 3, 2759 (2013). 24067913 10.1038/srep02759 PMC3783890

[b7] Dobson I., Carreras B. A., Lynch V.E. & Newman D. E. Complex systems analysis of series of blackouts: cascading failure, critical points, and self-organization. Chaos 17, 026103 (2007). 17614690 10.1063/1.2737822

[b8] Oppenheimer D., Ganapathi A. & Patterson D. A. Why do Internet services fail, and what can be done about it? In: Proc. 4th Usenix Symposium on Internet Technologies and Systems USITS'03, Seattle: USENIX Association, 1–16 (2003).

[b9] Atalay E., Hortasu A., Roberts J. & Syverson C. Network structure of production. P. Natl. Acad. Sci. USA 108, 5199–52102 (2011). 10.1073/pnas.1015564108 PMC3069152 21402924

[b10] Gai P. & Kapadia S. Contagion in financial networks. P. R. Soc. A 466, 2401–2423 (2010).

[b11] Haldane AG, May RM Systemic risk in banking ecosystems. Nature 469, 351–355 (2011). 21248842 10.1038/nature09659

[b12] Battiston S,, Puliga M,, Kaushik R,, Tasca P. & Caldarelli G. Debtrank: too central to fail? financial networks, the fed and systemic risk. Sci. Rep. 2, 541 (2012). 22870377 10.1038/srep00541 PMC3412322

[b13] Battiston S., Caldarelli G., Georg C. P., May R. & Stiglitz J. Complex derivatives. Nat. Phys. 9, 123–125 (2013).

[b14] Huang X., Vodenska I., Havlin S. & Stanley H.E. Cascading failures in bi-partite graphs: model for systemic risk propagation. Sci. Rep. 3, 1219 (2013). 23386974 10.1038/srep01219 PMC3564037

[b15] Schmitz O. J., Hambäck P. A. & Beckerman A. P. Trophic cascades in terrestrial systems: a review of the effects of carnivore removals on plants. Am. Nat. 155, 141–153 (2000). 10686157 10.1086/303311

[b16] Vespignani A. Complex networks: the fragility of interdependency. Nature 464, 984–985 (2010). 20393545 10.1038/464984a

[b17] Helbing D. Globally networked risks and how to respond. Nature 497, 51–59 (2013). 23636396 10.1038/nature12047

[b18] Diamond J. Collapse: How Societies Choose To Fail Or Survive (Penguin Books, 2004).

[b19] World Economic Forum Global Risks Report. (2013) Available at: http://www.weforum.org/reports/global-risks-2013-eighth-edition. (Accessed: 22nd March 2013)

[b20] World Economic Forum. (2013) Available at: http://www3.weforum.org/tools/rrn/wef_grr/20130108/server/getrisks.json. (Accessed: 12th April 2013)

[b21] Kaufman G. G. & Scott K. E. What is systemic risk, and do bank regulators retard or contribute to it? Indep. Rev. 7, 371–391 (2003).

[b22] Holland P. W., Laskey K. B. & Leinhardt S. Stochastic blockmodels: first steps. Soc. Networks 5, 109–137 (1983).

[b23] Cox D. R. & Miller H. D. The Theory Of Stochastic Processes (Methuen, 1965).

[b24] Beichelt F. Stochastic Processes In Science, Engineering And Finance (CRC Press, 2006).

[b25] Dempster A. P., Laird N. M. & Rubin D. B. Maximum likelihood from incomplete data via the em algorithm. J. R. Stat. Soc. B 1977:1–38 (1977).

[b26] Saez E. Striking it richer: the evolution of top incomes in the united states. (2013) Availbale at: http://eml.berkeley.edu/~saez/saez-UStopincomes-2012.pdf. (Accessed: 12th July 2014)

[b27] Pawitan Y. In All Likelihood: Statistical Modelling And Inference Using Likelihood (Oxford Science Publications Clarendon Press, 2001).

[b28] Cole N *et al.* Maximum likelihood fitting of tidal streams with application to the sagittarius dwarf tidal tails. Astrophys. J. 683, 750–766 (2008).

[b29] Mandela D. R. & Barnesb A. Accuracy of forecasts in strategic intelligence. P. Natl. Acad. Sci. USA 111, 10984–10989 (2014). 10.1073/pnas.1406138111 PMC4121776 25024176

